# The Use of the Microscope in Progressive Dentistry

**Published:** 1887-11

**Authors:** L. L. Davis


ARTICLE VII.

### THE USE OF THE MICROSCOPE IN PROGRESSIVE DENTISTRY.

BY L. L. DAVIS, D. D. S.

The microscope has long been regarded as a mere toy, capable of affording amusement only, but its importance and usefulness grows every year, and there seems no limit to the extent of its services in all professions and trades. While it pours out its greatest tribute to the medical profession, yet druggists, merchants, bankers and lawyers all claim its valuable assistance. One detects drug adulterations, another examines the threads of the fabrics offered for sale, or tests the genuineness of a signature.

The dentist, too, has need of its aid, and it is for the purpose of showing those needs this paper is written.

To-day there is no instrument in existence that can vie with it in the discoveries made by its use, which have helped the cause of science. To-day the men of whom the masses speak the most are men whose labors with this instrument have brought them into public notice. Even ignorant and unlettered people have heard of Pasteur and Koch, and what dentist has not heard of Black and Miller, Abbott and Bœdeker, Sudduth and other prominent men in the profession who give this study their special attention?

By means of the microscope man has unveiled the mysteries of nature to which he has so long been blind, and new worlds are now open to research, and permanent fame awaits the earnest seekers after knowledge in this attractive field of study.

The present importance of this instrument warrants the assertion that is to become one of the prime educators of ensuing generations.

Anatomy with all its attractions and splendid prospects is left far behind in the race for highest knowledge of the human form.

The surgeon who looks in your face and sees not only the lines, angles, prominences and depressions which form the features, but traces beneath the surface-tissue the nerves, arteries and arrangement and attachment of the muscles to the bony frame-work, may feel a worthy pride in his greater knowledge. But what words of mine can express the feelings of the microscopist as he takes that nerve, that artery or that muscles and resolves it into the most minute and primitive cell structure, and arranging and classifying each tissue component that are the genesis of that highly organized being, man, till he is almost able to *grasp* the vital principal called life.

The cry for post-graduate study has gone forth and an able scheme or schedule for such study has been prepared by Dr. Moody, yet could I so influence the profession, I would have every man with microscope in hand as a necessary adjunct to all his future work.

To whom does dentistry owe its greatest honor? to the mechanic with his wonderful skill in restoring lost organs; to the operator in repairing the ravages of decay, or to those men who, by their painstaking study have placed within our power the means to *prevent* decay?

Inoculation of a mild form of virus has made it possible to withstand the most horrible of all diseases. The germ theory has received such strong indorsement from all thinking men that it stands firmly and resists the puny attacks of those who, from want of education or their natural surroundings, can only believe what is possible to them, and thus give us a "bug" theory.

You are all familiar enough with Dr. Black's experiments in cultivating germs or fungi to be forced to acknowledge that something does develop from a very small beginning, and the microscope shows that small beginning to be a minute rod or ball, that has the property of reproducing its like in places favorable to such growth.

What blind seeking in the dark, what vague theorizing would arise had we not the microscope to aid us in the work.

The knowledge of physiology is essential to the study of pathology, and in dentistry the minute anatomy of tooth structures should be as familiar to the operator as the shape of each individual tooth, as also the changes in each structure by disease.

Only a few years ago the number of medical and dental colleges having the study of microscopy in their curicule could be counted on the fingers, but to such prominence has this instrument forced its way that to-day no college of repute dare slight the study. That a student should become so versed in histological study during the short time spent in college, as to prove an expert, is no more expected than is his studies in any other branch, but such a foundation should be laid, and the study made so attractive that his after life may prove the efficiency of the teaching.

The University of Michigan was among the first to recognize the importance of the microscope in medical study, and slowly but surely the other colleges followed in her wake, then the dental students were brought into action, and again the colleges responded.

The Chicago College of Dental Surgery has, from the beginning, recognized the value of this study, and by the stimulus of Prof. Black's teachings, is to-day the first and only dental and medical college in the world to have in its equipment a complete apparatus and laboratory for the furtherance of bacteriological investigation. While practical methods of filling teeth are of paramount importance to the dentist, there are those whose thoughts may give vent to such expressions as "What do I want to know about something I can't see without a spy-glass?" "Tell me how to fill teeth better, or make a better fitting plate, that's my want." To those practical men I say *you*, YOU are the men I am looking to to receive the benefit of this great study.

The use of antiseptics and disinfectants will aid you not only to be more sure of your operations on the natural organs, but will place within your reach the means of securing the best results from artificial substitutes. Then let me more earnestly impress upon you the importance of the microscope in your every-day practice, let your relaxation from professional duties find pleasant occupation in striving to discover more perfect theories for the causes of diseases, and your efforts bear fruitage in the advancement of this most wonderful of all instruments, the microscope.

'Tis not my intention to lay out for you the plan you should pursue in carrying on this study, nor am I here to discourse on the merits and demerits of the many text-books on this subject. Men in this, like all other fields of research, have ideas and theories of their own, and the one peculiar feature of this study is that *faith* is not the most essential qualifications for its progressive.

Let a man doubt every theory he reads till his own eyes and reason have compelled him to give assent to its truth or show its utter falsity.

To the young men of the profession let me say there is no field so wide in which to carve out name and fame; to the older members, weary with the strivings of a busy life, in no other line of intellectual development will you find so fascinating and pleasant occupation; and to those in the prime and vigor of manhood, it will prove a valuable ally in the good cause for which you are striving.

Excelsior then be the motto. Lift high the banner to the breeze, and as the moving army press onward and upward let each man make use of every aid to place his loved profession upon the pinnacle of fame.—*Transactions of the Illinois State Dental Society.*

